# Scoping review on the association between early childhood caries and responsible resource consumption and production: exploring Sustainable Development Goal 12

**DOI:** 10.1186/s12903-023-03831-0

**Published:** 2024-01-17

**Authors:** Morẹ́nikẹ́ Oluwátóyìn Foláyan, Jorma I. Virtanen, Balgis Gaffar, Olunike Abodunrin, Ivy Guofang Sun, Duangporn Duangthip, Arthur Kemoli, Ray M. Masumo, Ana Vukovic, Ola B. Al-Batayneh, Tshepiso Mfolo, Robert J Schroth, Maha El Tantawi

**Affiliations:** 1 Early Childhood Caries Advocacy Group, Winnipeg, Canada; 2 Department of Child Dental Health, Obafemi Awolowo University, Ile-Ife, Nigeria; 3 Faculty of Medicine, University of Bergen, Bergen, Norway; 4 Department of Preventive Dental Sciences, College of Dentistry, Imam Abdulrahman Bin Faisal University, Dammam, Saudi Arabia; 5 Lagos State Health Management Agency, Lagos, Nigeria; 6 Faculty of Dentistry, The University of Hong Kong, Hong Kong SAR, China; 7 Department of Paediatric Dentistry and Orthodontics, University of Nairobi, Nairobi, Kenya; 8 Department of Community Health and Nutrition, Tanzania Food and Nutrition Centre, Dar es Salaam, Tanzania; 9 Clinic for Pediatric and Preventive Dentistry, School of Dental Medicine, University of Belgrade, Belgrade, Serbia; 10 Department of Orthodontics, Pediatric and Community Dentistry, College of Dental Medicine, University of Sharjah, Sharjah, United Arab Emirates; 11 Department of Preventive Dentistry, Faculty of Dentistry, Jordan University of Science and Technology, Irbid, Jordan; 12 Department of Community Dentistry, University of Pretoria, Hatfield, South Africa; 13 Dr. Gerald Niznick College of Dentistry, Rady Faculty of Health Sciences, University of Manitoba, Winnipeg, Canada; 14 Department of Pediatric Dentistry and Dental Public Health, Faculty of Dentistry, Alexandria University, Alexandria, Egypt

**Keywords:** Sustainable development, Dental waste, Dental caries, Child, Preschool, Waste minimization, Sustainable consumption, resource consumption, resource utilization maximization, responsible consumption, minimum intervention dentistry

## Abstract

**Background:**

The Sustainable Development Goal 12 (SDG12) promotes patterns that minimize waste and maximize resource utilization. It is therefore plausible that preventing Early Childhood Caries (ECC) and promoting oral health can contribute to sustainable consumption. In addition, sustainable consumption and production can contribute to the control of ECC. This scoping review aimed to explore the possible evidence on the link between ECC and the SDG12 targets.

**Methods:**

This scoping review identified articles on the link between resource consumption and production and caries according to the PRISMA-ScR guidelines. Three electronic databases (PubMed, Web of Science, and Scopus) were systematically searched in August 2023, using specific search terms. Studies written in English, with full text available, addressing dental caries and linked with waste minimization and resource utilization maximisation, with results that could be extrapolated to ECC in children less than 6 years of age) were included. Descriptive statistics were planned to summarize the categories of retrieved papers.

**Results:**

The initial search yielded 904 articles, with 863 screened for eligibility after the removal of duplicates. No studies were identified that reported data on an association between responsible consumption and production of resources factors and ECC.

**Conclusion:**

This scoping review did not identify any articles published in English on evidence of the direct associations between ECC and SDG12 targets. However, there is a plausibility of such a link using minimum intervention dentistry for ECC management as a waste prevention and resource utilisation maximization strategy.

**Supplementary Information:**

The online version contains supplementary material available at 10.1186/s12903-023-03831-0.

## Introduction

The Sustainable Development Goal 12 (SDG12) has the objective of establishing sustainable patterns of consumption and production of resources [1]. It calls for a comprehensive range of actions involving businesses (SDG 12.6), policymakers (12.7), and consumers (12.8) to adopt sustainable practices [2]. The goal envisions sustainable resource production and consumption based on advanced technology, resource efficiency, and reduced global waste [2] since consumption and production patterns have significant environmental and social implications.

A strategic action taken to achieve this goal involves decoupling economic growth from resource utilization [3]. Although economic growth enhances people’s well-being, it has traditionally relied on increasing resource and energy consumption [4]. The continuous escalation of finite resource consumption harms the environment [4]. To address this challenge, economic growth must prioritize enhancing resource and energy efficiency by restructuring economies to achieve more output from the same resource and energy inputs. In the context of sustainable consumption, SDG12 promotes patterns that minimize waste and maximize resource utilization [5, 6].

Preventing Early Childhood Caries (ECC), which is tooth decay in the primary teeth of children less than 6 years of age [7], can contribute to sustainable consumption by reducing the need for extensive rehabilitative dental treatment, such as restorations or extractions, which require substantial amounts of resources, including materials, energy, and water [8, 9]. It can also reduce the waste generated within dental practices [10]. In addition, efforts geared at keeping materials and resources in the economy for as long as possible, through repair, recycling and reuse, and minimising or preventing waste can help with achieving the targets of the SDG12 [11, 12].

The high global burden of ECC makes it important to reflect on how the management of ECC may contribute to the attainment of the SDG12. There are currently, 514 million children living with ECC [13]. This will need a significant quantity of dental materials to manage these carious lesions. In addition, because ECC affects the primary teeth, and primary teeth exfoliate, restored primary teeth may become environmental contaminants. Therefore, thinking critically and developing environmentally friendly restorative materials for use in the primary dentition can strategically help maximize safe resource use. Such materials should be less likely to produce toxic wastes and should not be a pollutant of the environment.

**Fig. 1:**
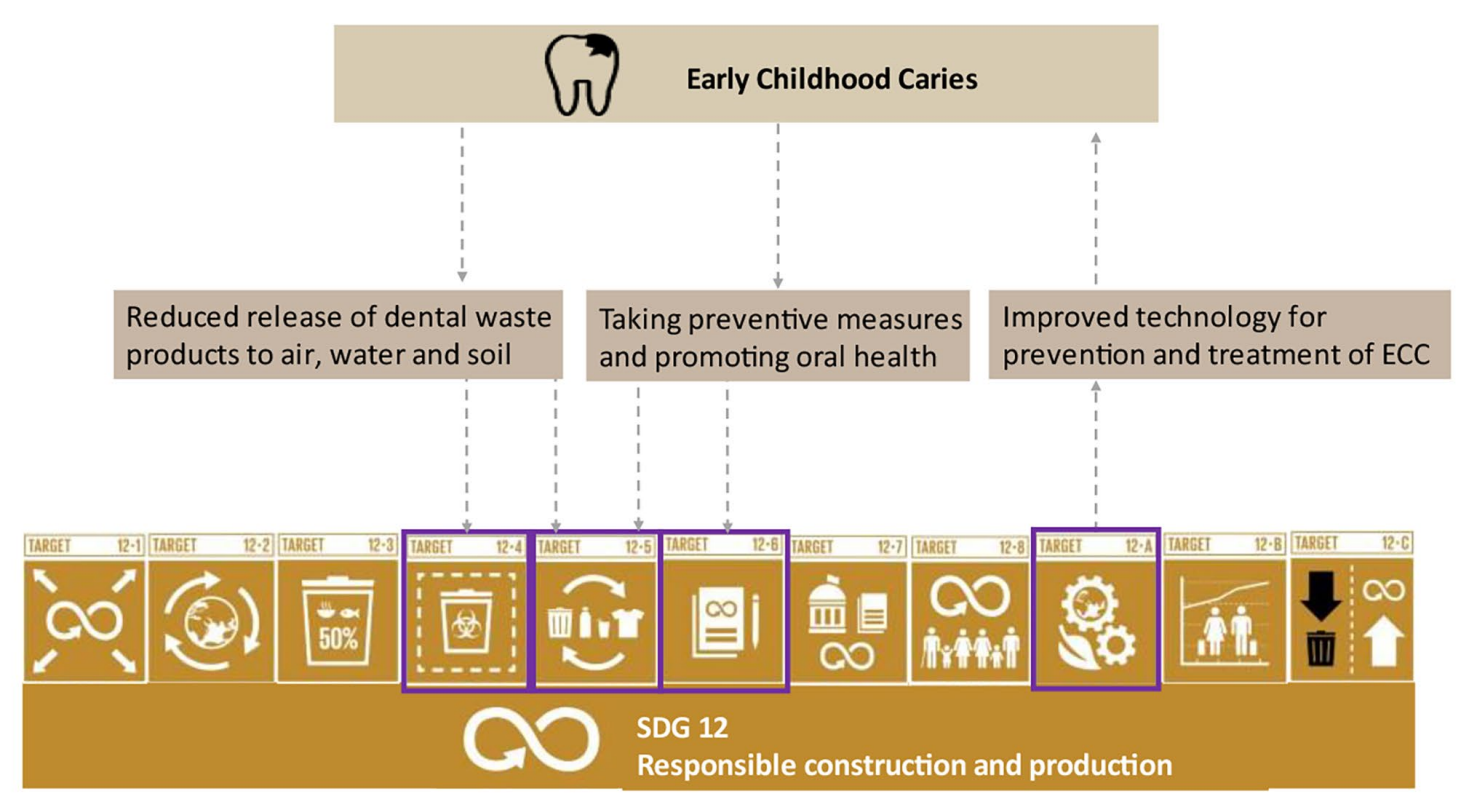
The conceptual framework of ECC and responsible resource consumption and production (SDG12). (12 − 4 Responsible management of chemicals and waste. 12 − 5 Substantially reduce waste generation. 12 − 6 Encourage companies to adopt sustainable practices and sustainability reporting. 12 − A Support developing countries’ scientific and technological capacity for sustainable consumption and production)

SDG12 comprises 11 targets, one of which is the SDG12.4 that aims to promote environmentally sound management of chemicals and wastes throughout their life cycle to minimize adverse impacts on human health and the environment. Evidence shows that dental caries waste and dental products released into the environment can have harmful effects [14–17]. The annual waste generated from dental clinics also contributes to environmental pollution [18]. Thus, effective ECC control may lead to a substantial reduction in waste generation through prevention, reduction, recycling, and reuse, supporting the achievement of SDG12.5 [19]. The adoption of sustainable practices by dental hospitals and clinics, along with reporting on their sustainability efforts, can contribute to SDG12.6. Focusing on developing countries, which currently bear a higher burden of untreated ECC, aligns with SDG12.A and can harness the potential of SDG12 for ECC control. Obtaining specific information on the link between ECC management, effective waste management, and resource savings may encourage investment in caries prevention efforts for preschool children. The conceptual framework illustrating the association between SDG12 and ECC is depicted in Fig. 1.

Currently, investment in ECC management is low, as there is a common assumption that primary teeth will naturally exfoliate [20]. However, untreated ECC has both short- and long-term negative impacts on the growth, development, general health and well-being, quality of life of the affected children and their parents [21–23] as well as the future health of the permanent dentition [24]. Since children affected by ECC are in the pre-cooperative period, treatment often requires sedation or general anaesthesia, leading to significant costs for families [25] in addition to the huge generation of wastes from the use of tubes, anaesthesia gases and hospital laundry amongst others [26]. This places a considerable burden on existing resources, necessitating budgetary allocations for specialized care [27], Investing in ECC control could yield substantial benefits for the growth, development, health, well-being, and quality of life of infants, toddlers, and preschool children, while also contributing to environmental health and the objectives of SDG12. The purpose of this scoping review was to identify the possible links between ECC and SDG12 targets.

## Methods

A scoping review was performed to investigate the association between ECC and SDG12. The review adhered to the Preferred Reporting Items for Systematic Reviews and Meta-Analyses Extension for Scoping Reviews (PRISMA-ScR) guidelines [28] to ensure methodological rigor and transparency.

### Research questions

The following questions guided this review: (1) What is the existing evidence on how responsible consumption and production of resources affect and are affected by ECC; (2) What are the responsible consumption and production related factors (disposal of toxic waste and pollutants, recycle and reduce waste, per capita global food waste at the retailer and consumer levels) linked to ECC?

### Articles identification

The initial search was conducted on three electronic databases PubMed, Web of Science and Scopus in August 2023. The search was performed using the pre-generated query string for the SDG 12 presented in the advanced search function of Scopus [29] shown in Appendix 1. Search terms were tailored to the specific requirements of each database. The search was completed in August 2023. No protocol was published for this review.

### Eligibility and selection

Literature obtained through database searches was exported to the reference management Rayyan, where duplicates were removed using the “duplicate items” function. Title and abstract screening were conducted by two independent reviewers (OA and MOF), guided by eligibility criteria for this review. Full-text review of the extracted publication was then completed independently by two researchers (OA and MOF). Resolutions of conflicts in manuscript selection in was resolved through consultation with one of the authors (MET). No attempts were made to contact authors or institutions to find additional sources. Any published manuscript presenting findings related to the association between oral health and the SDG12. Inclusion criteria required that the publications be in English and have full texts available for extracting all relevant information. The review included letters, reviews, conference reports, observational studies, and experimental studies while excluding books, and grey literature publications. No authors or institutions were contacted to identify additional sources.

### Inclusion criteria

This review only included English language publications. Studies that presented findings about the association between consumption, production, toxic waste, pollutants, recycle waste, reduce waste and ECC among children aged 71 months and below were included in the review.

### Exclusion criteria

Studies focusing on ECC prevalence only without reporting risk factors were excluded from this review.

### Data charting

From the publications included in this review, the following data were planned to be extracted: author, publication year, study location, study design, study sample size and age of the children, study aim, and main findings. The extracted information from each publication was planned to be compiled and summarized into one table.

## Results

**Fig. 2:**
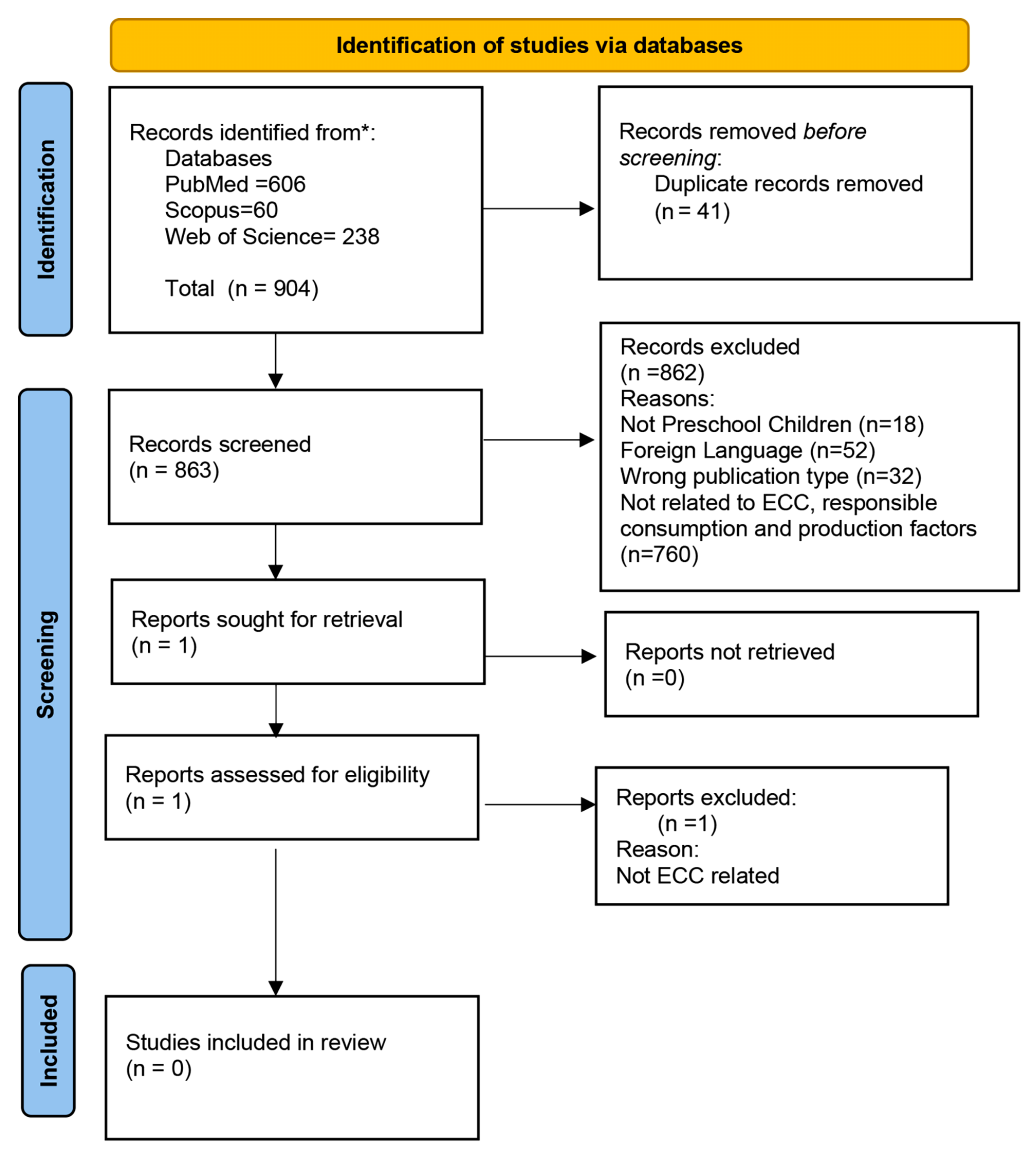
Flowchart on studies identified during the systematic search of the literature on the link between SDG12 and ECC

The initial search using the predefined search terms from the three databases (PubMed, Web of Science and Scopus) yielded 904 articles. After duplicates removal 863 were screened for eligibility. No studies were identified that reported data on the association between responsible consumption and production factors and ECC. The search flowchart is depicted in Fig. 2.

The single article assessed for eligibility during the screening phase was authored by Drummond et al. and published in 2003 [30]. A systematic assessment of six dental offices or clinics was conducted over a 12–18-month period with the aim of assessing the generation and disposal of amalgam waste in dental practices. The study found that the generation rate of non-contact amalgam (unused material) ranged from 0 to 1672 mg/day/chair, with a median value of 421 mg/day/chair. The contact amalgam generation rate (used in tooth restoration and later removed) ranged from 9 to 466 mg/day/chair, with a median value of 64 mg/day/chair. Overall, the study highlighted the variability in amalgam waste generation among dental practices and provided estimates for the potential environmental impact of such waste at both the State of Illinois and the United States of America.

## Discussion

This scoping review did not identify any published evidence in the English literature on the association between ECC and SDG12, despite indications that such a link is plausible. Trying to make a direct connection between ECC and SDG12 may necessitate a broader perspective of oral health care [11]. Taking preventive measures and promoting oral health can contribute to the overarching sustainability agenda by reducing resource consumption and improving health and well-being.

This scoping review represents the first comprehensive analysis examining the potential association between ECC the SDG12. This contributes to bridging the existing gap in literature regarding the specific link between responsible resource consumption and production, and the oral health of children. The study highlights the need for further investigation and evidence generation on the link between SDG12 and ECC. Despite these strengths, this scoping review has some limitations. The present scoping review is limited to publications in English. This may have resulted in missed resources generated in the 52 search results written in other languages. In addition, the retrieved publications were mapped against the pre-generated query string presented by Scopus and thus, although adding specific dental terms to the strings may retrieve additional results, this approach may affect the consistency of findings and ability to compare results to non-dental context.

Despite the lack of publications on the link between ECC and the SDG12, this association is plausible. The use of recycled dental materials such as glass ionomer cements for the management of ECC [31], will contribute to achieving the SDG12. Dental materials such as glass ionomer cements, can also be made from recycled products [32]. Bioresorbable and biodegradable materials, including biodegradable bioceramics and polymers, can also be utilized in the production of prosthetic dental materials [33]. Furthermore, paper, and plastic autoclave bags can be substituted with compostable bags [34]. Reusable sterilization pouches [35], face shields, air/water syringes, and impression trays are also available alternatives [36].

In addition, dental clinics can also opt to recycle outdated or unneeded dental instruments [36]. Embracing reusable alternatives can lead to significant environmental benefits for a solo practitioner, diverting up to 4680 paper and plastic autoclave bags each year [37]. Also, adopting reduce, reuse, recycle principles for plastic consumables and introducing sustainable procurement procedures could lead to reduction in the estimated 388 tonnes of CO² emission attributed to staff travel, business travel and procurement; and an estimated 1 million plastic items distributed while caring for children [38].

One significant development in the dental field is the transition from amalgam to tooth-coloured restorative materials. Over the last decade, this shift has been largely driven by the Minamata Convention on Mercury, adopted by delegates from over 140 countries on January 19, 2013, after three years of negotiations [39]. The focus of advocacy efforts to eliminate mercury use in dentistry has primarily been on environmental safety concerns and its connection to SDG12 [40]. The use of tooth-coloured restorative materials is also an environmental safety movement that promotes substantial waste reduction through prevention, reduction, recycling, and reuse, thereby contributing to the achievement of SDG 12 [41, 42].

Tooth-coloured materials play a role in resource efficiency and waste reduction. Unlike traditional amalgam fillings, which require a larger amount of material to fill cavities, leading to increased resource consumption, tooth-coloured materials allow for more conservative preparations, minimizing the removal of healthy tooth structure during the restorative process [43]. This preservation of natural tooth structure not only contributes to better oral health but also reduces material consumption. Furthermore, tooth-coloured restorations can be repaired rather than replaced, further minimizing waste generation, and promoting sustainable production practices [10]. These restorations also exhibit a lower environmental impact throughout their life cycle, including reduced energy consumption and carbon emissions during production and a smaller ecological footprint during disposal [10]. By opting for tooth-coloured materials, dental professionals actively support sustainable production practices that prioritize environmental preservation [41, 42, 44, 45].

A search of the literature between 2015 and 2023, however, identified no studies that explicitly explored links between SDG12 and oral health [46]. The complexity of interactions among various SDGs that influences the prevalence, burden, and severity of oral health may pose a challenge in isolating the specific impact of SDG12 on ECC. These underscore the need for more primary studies, empirical evidence, and collaborative efforts to measure he intricate relationship between responsible resource consumption and production, and the oral health of children as the efforts of the dental industry to contribute to SDG12 are particularly crucial in addressing ECC.

A public endorsement of the practice of green dentistry can promote responsible resource consumption and production [36], including considerations for reducing the risk for ECC, and eliminating untreated ECC. Untreated ECC increases the risk of caries in permanent teeth [46], implying that many teeth may require restorative materials for their entire lifespan. Effectively controlling ECC can contribute to managing the use of dental materials for tooth restoration, thereby reducing dental waste. Additionally, the use of tooth-coloured materials like glass ionomer cements contributes to reducing the risk of secondary caries and caries in permanent teeth [47], which in turn reduces the need for lifelong restorative materials. The Children’s Amendment of the Minamata Convention on Mercury that promotes reduction of mercury from amalgam use can actively contribute the attainment of the SDG12 [48]. These possibilities, however, warrant empirical exploration to further understand their impact.

One way to generate supportive evidence is to actively monitor the contribution of mercury free management of ECC to the SDG12. A lead can be taken from the active monitoring of the progress made by the European Union towards achieving the SDGs [49]. There are evidence on measures for assessing waste generation that can be adapted for measuring the impact of SDG12 through the management of ECC and or waste reduction through the implementation of ECC control plans [50]. This may require that countries that signed to the Minamata Convention on Mercury contribute national data to this monitoring platform to assess progress with the sustainability agenda of the SDG as a whole and the SDG12 specifically.

Measuring the connections between ECC and the SDGs is crucial for targeted investment. An example from the European Union’s monitoring of SDG12 reveals that 4.4% of total waste generated in 2020 posed health or environmental hazards, highlighting the importance of effective management of hazardous chemical-laden industrial waste [50]. While these adverse effects are most acute near production facilities, chemical waste often contaminates water sources, oceans, and soil, affecting crops that are traded and consumed globally [51]. A concerning illustration is the presence of “super-bugs” in water bodies near antibiotic production plants, which are resistant to antimicrobials, posing a global health risk [51]. Such findings inform the development of policies and programs to enhance overall health, and with specific ECC related information, can help improve oral health including ECC.

In conclusion, although there is currently no published evidence in the English literature on the direct associations between ECC and the SDG12, this link is plausible and can be explored empirically. Such data will likely promote investment in the principles of the Minamata Convention on Mercury as well as investment in ECC management.

### Electronic supplementary material

Below is the link to the electronic supplementary material.

Supplementary Material 1

## Data Availability

All data generated or analysed during this study are included in this published article.

## Abbreviations

ECC: Early Childhood Caries
PRISMA-ScR: Preferred Reporting Items for Systematic Reviews and Meta-Analyses Extension for Scoping Reviews guidelines
SDG: Sustainable Development Goal
