# Supplementary material for: Scoping review on the association between early childhood caries and responsible resource consumption and production: exploring Sustainable Development Goal 12
Source: BMC Oral Health. 2024 Jan 17;24:98. doi: 10.1186/s12903-023-03831-0 (PMC10792892; doi:10.1186/s12903-023-03831-0)
Supplement: Supplementary file 1 — Supplementary Material 1 [file 12903_2023_3831_MOESM1_ESM.docx]

Appendix 1

Search strategy for Scopus

| # | Search Terms | Results |
| --- | --- | --- |
| 3 | ( TITLE-ABS-KEY ( {environmental pollution}  OR  {hazardous waste}  OR  {hazardous chemical}  OR  {hazardous chemicals}  OR  {toxic chemical}  OR  {toxic chemicals}  OR  {chemical pollution}  OR  {ozone depletion}  OR  {pesticide pollution}  OR  {pesticide stress}  OR  {pesticide reduction}  OR  {life cycle assessment}  OR  {life cycle analysis}  OR  {life cycle analyses}  OR  {life-cycle analysis}  OR  {life-cycle analyses}  OR  {low carbon economy}  OR  {low-carbon economy}  OR  {environmental footprint}  OR  {material footprint}  OR  {harvest efficiency}  OR  {solid waste}  OR  {waste generation}  OR  {corporate social responsibility}  OR  {corporate sustainability}  OR  {consumer behavior}  OR  {consumer behaviors}  OR  {consumer behaviour}  OR  {consumer behaviours}  OR  {waste recycling}  OR  {resource recycling}  OR  {resource reuse}  OR  {biobased economy}  OR  {zero waste}  OR  {sustainability label}  OR  {sustainability labelling}  OR  {global resource extraction}  OR  {material flow accounting}  OR  {societal metabolism}  OR  {food spill}  OR  {resource spill}  OR  {resource efficiency}  OR  {sustainable food consumption}  OR  {green consumption}  OR  {sustainable supply chain}  OR  {circular economy}  OR  {cradle to cradle}  OR  {sustainable procurement}  OR  {sustainable tourism}  OR  {fossil-fuel subsidies}  OR  {fossil-fuel expenditure}  OR  {sustainability label}  OR  {sustainability labelling}  OR  ( consumption  AND  ( {resource use}  OR  spill ) )  OR  ( production  AND  ( {resource use}  OR  spill ) )  AND NOT  ( {wireless sensor network}  OR  {wireless sensor networks}  OR  {wireless network}  OR  {wireless networks}  OR  {wireless}  OR  {disease}  OR  {astrophysics} ) ) )  AND  ( TITLE-ABS-KEY ( caries )  OR  TITLE-ABS-KEY ( dental  AND  caries )  OR  TITLE-ABS-KEY ( dental  AND  decay )  OR  TITLE-ABS-KEY ( dental  AND  cavities )  OR  TITLE-ABS-KEY ( enamel  AND  demineralization )  OR  TITLE-ABS-KEY ( tooth  AND  demineralization )  OR  TITLE-ABS-KEY ( tooth  AND  cavities ) ) View Less | [60 document results](https://08105f8j7-1105-y-https-www-scopus-com.mplbci.ekb.eg/search/history/results.uri?origin=searchhistory&shid=3) |
| 2 | TITLE-ABS-KEY ( caries )  OR  TITLE-ABS-KEY ( dental  AND  caries )  OR  TITLE-ABS-KEY ( dental  AND  decay )  OR  TITLE-ABS-KEY ( dental  AND  cavities )  OR  TITLE-ABS-KEY ( enamel  AND  demineralization )  OR  TITLE-ABS-KEY ( tooth  AND  demineralization )  OR  TITLE-ABS-KEY ( tooth  AND  cavities ) | [125,305 document results](https://08105f8j7-1105-y-https-www-scopus-com.mplbci.ekb.eg/search/history/results.uri?origin=searchhistory&shid=2) |
| 1 | TITLE-ABS-KEY ( {environmental pollution}  OR  {hazardous waste}  OR  {hazardous chemical}  OR  {hazardous chemicals}  OR  {toxic chemical}  OR  {toxic chemicals}  OR  {chemical pollution}  OR  {ozone depletion}  OR  {pesticide pollution}  OR  {pesticide stress}  OR  {pesticide reduction}  OR  {life cycle assessment}  OR  {life cycle analysis}  OR  {life cycle analyses}  OR  {life-cycle analysis}  OR  {life-cycle analyses}  OR  {low carbon economy}  OR  {low-carbon economy}  OR  {environmental footprint}  OR  {material footprint}  OR  {harvest efficiency}  OR  {solid waste}  OR  {waste generation}  OR  {corporate social responsibility}  OR  {corporate sustainability}  OR  {consumer behavior}  OR  {consumer behaviors}  OR  {consumer behaviour}  OR  {consumer behaviours}  OR  {waste recycling}  OR  {resource recycling}  OR  {resource reuse}  OR  {biobased economy}  OR  {zero waste}  OR  {sustainability label}  OR  {sustainability labelling}  OR  {global resource extraction}  OR  {material flow accounting}  OR  {societal metabolism}  OR  {food spill}  OR  {resource spill}  OR  {resource efficiency}  OR  {sustainable food consumption}  OR  {green consumption}  OR  {sustainable supply chain}  OR  {circular economy}  OR  {cradle to cradle}  OR  {sustainable procurement}  OR  {sustainable tourism}  OR  {fossil-fuel subsidies}  OR  {fossil-fuel expenditure}  OR  {sustainability label}  OR  {sustainability labelling}  OR  ( consumption  AND  ( {resource use}  OR  spill ) )  OR  ( production  AND  ( {resource use}  OR  spill ) )  AND NOT  ( {wireless sensor network}  OR  {wireless sensor networks}  OR  {wireless network}  OR  {wireless networks}  OR  {wireless}  OR  {disease}  OR  {astrophysics} ) ) View Less | [351,469 document results](https://08105f8j7-1105-y-https-www-scopus-com.mplbci.ekb.eg/search/history/results.uri?origin=searchhistory&shid=1) |

Search strategy for WOS

- WOS.SCI: 1900 to 2023

- WOS.AHCI: 1975 to 2023
- WOS.BHCI: 2005 to 2023
- WOS.BSCI: 2005 to 2023
- WOS.ESCI: 2005 to 2023
- WOS.ISTP: 1990 to 2023
- WOS.SSCI: 1900 to 2023
- WOS.ISSHP: 1990 to 2023

| # | Search Query | Results |
| --- | --- | --- |
| 1 | ((((((((((((((((((((((((((((((((((((((((TS=(environmental pollution)) OR TS=(hazardous waste)) OR TS=(hazardous chemical)) OR TS=(toxic chemical)) OR TS=(chemical pollution)) OR TS=(ozone depletion)) OR TS=(pesticide pollution)) OR TS=(pesticide stress)) OR TS=(pesticide reduction)) OR TS=(life cycle assessment)) OR TS=(life cycle analysis)) OR TS=(low carbon economy)) OR TS=(environmental footprint)) OR TS=(material footprint)) OR TS=(harvest efficiency)) OR TS=(solid waste)) OR TS=(waste generation)) OR TS=(corporate social responsibility)) OR TS=(corporate sustainability)) OR TS=(consumer behavior)) OR TS=(resource recycling)) OR TS=(resource reuse)) OR TS=(biobased economy)) OR TS=(zero waste)) OR TS=(sustainability label)) OR TS=(sustainability labelling)) OR TS=(global resource extraction)) OR TS=(material flow accounting)) OR TS=(societal metabolism)) OR TS=(food spill)) OR TS=(resource spill)) OR TS=(resource efficiency)) OR TS=(sustainable food consumption)) OR TS=(green consumption)) OR TS=(sustainable supply chain)) OR TS=(circular economy)) OR TS=(cradle to cradle)) OR TS=(sustainable procurement)) OR TS=(sustainable tourism)) OR TS=(fossil-fuel subsidies)) OR TS=(fossil-fuel expenditure) | 960451 |
| 2 | ((((((TS=(caries)) OR TS=(dental caries)) OR TS=(dental decay)) OR TS=(dental cavities)) OR TS=(tooth cavities)) OR TS=(tooth deminerali?ation)) OR TS=(enamel deminerali?ation) | 67522 |
| 3 | #2 AND #1 | 238 |

Search strategy for Pubmed

| Search number | Query | Results |
| --- | --- | --- |
| 5 | #3 AND #4 | 606 |
| 4 | (((((("Dental Caries"[Mesh]) OR "Tooth Demineralization"[Mesh]) OR (caries[Text Word])) OR (dental decay[Text Word])) OR (dental cavities [Text Word])) OR (tooth cavities[Text Word])) OR (enamel demineralization[Text Word]) | 71,618 |
| 3 | #1 OR #2 | 691,134 |
| 2 | ((((((((((((((((((((((((((((((((((toxic chemical[Text Word]) OR (chemical pollution[Text Word])) OR (pesticide pollution[Text Word])) OR (pesticide stress[Text Word])) OR (pesticide reduction[Text Word])) OR (life cycle assessment[Text Word])) OR (life cycle analysis[Text Word])) OR (low carbon economy[Text Word])) OR (environmental footprint[Text Word])) OR (material footprint[Text Word])) OR (harvest efficiency[Text Word])) OR (waste generation[Text Word])) OR (corporate social responsibility[Text Word])) OR (corporate sustainability[Text Word])) OR (resource recycling[Text Word])) OR (resource reuse[Text Word])) OR (biobased economy[Text Word])) OR (zero waste[Text Word])) OR (sustainability label[Text Word])) OR (sustainability labelling[Text Word])) OR (global resource extraction[Text Word])) OR (material flow accounting[Text Word])) OR (societal metabolism[Text Word])) OR (food spill[Text Word])) OR (resource spill[Text Word])) OR (resource efficiency[Text Word])) OR (sustainable food consumption[Text Word])) OR (green consumption[Text Word])) OR (sustainable supply chain[Text Word])) OR (circular economy[Text Word])) OR (cradle to cradle[Text Word])) OR (sustainable procurement[Text Word])) OR (sustainable tourism[Text Word])) OR (fossil-fuel subsidies[Text Word])) OR (fossil-fuel expenditure[Text Word]) | 17,281 |
| 1 | (((((("Environmental Pollution"[Mesh]) OR "Hazardous Waste"[Mesh]) OR "Hazardous Substances"[Mesh]) OR "Ozone Depletion"[Mesh]) OR "Solid Waste"[Mesh]) OR "Consumer Behavior"[Mesh]) OR "Resource Allocation"[Mesh] | 678,657 |
